# New records of chalcidid (Hymenoptera: Chalcididae) pupal parasitoids from India

**DOI:** 10.3897/BDJ.4.e6900

**Published:** 2016-01-21

**Authors:** Prakash Gowri, Sagadai Manickavasagam, Rasappan Kanagarajan

**Affiliations:** ‡ Annamalai University, Faculty of Agriculture, Chidambaram, India; § Annamalai University, Department of Entomology, Chidambaram, Tamil Nadu, India

**Keywords:** Insecta, Chalcidoidea, Distributional records, India

## Abstract

**Background:**

Chalcidids are one of the most important parasitoids of pupae of agriculturally important pests belonging to orders like Lepidoptera, Diptera, Coleoptera and Hymenoptera. Such an important group has not been studied consistently by any team of workers from any country apart from the notable contributions by Boucek, Steffan, Delvare and Narendran. (Boucek 1988, Steffan 1973, Delvare 1992 and Narendran 1989). On a personal note, Dr. John S Noyes of Natural History Museum London agrees with this view as expressed with the second author and hence we felt that we can initiate further work on this group within India. We currently hold hundreds of unidentified specimens of this family in our department collection confirming that we will have much work to do over a long period of time.

**New information:**

New distribution records of Chalcididae from Andhra Pradesh (*Brachymeria megaspila*, *B. minuta*, *Dirhinus anthracia* and *D. auratus*), Bihar (*B. podagrica*, *B. excarinata*, *B. hearseyi*, *D. anthracia*, *D. auratus*, *D. pilifer*, *Epitranus erythrogaster* and *Psilochalcis carinigena*), Karnataka (*B. apicicornis*), Manipur (*B. euploeae*, *D. auratus* and *E. erythrogaster*), Mizoram (*B. euploeae* and *D. anthracia*), Nagaland (*B. euploeae*), Himachal Pradesh (*B. alternipes*), and Tamil Nadu (*B. apicicornis*, *D. anthracia*, *D. deplanatus*, *D. pilifer*, *D. bakeri*, *E. observator*, *E. elongatulus*, *P. keralensis* and *P. soudanensis*) and union territories Andaman & Nicobar Islands (*B. podagrica*, *B. excarinata*, *E. erythrogaster* and *P. carinigena*) and Pudhucherry (*B. albicrus*, *D. anthracia*, *D. auratus*, *E. erythrogaster* and *P. kerelensis*) are documented from the unidentified material mentioned above.

## Introduction

Members of the family Chalcididae are predominantly primary endoparasitoids of Lepidoptera and Diptera, though a few species attack Hymenoptera, Coleoptera and Neuroptera (Gibson 1996). Though there are about 90 genera and 1530 species globally, only 29 genera and 214 species are reported from India (Noyes 2015) indicating that the Indian fauna is poorly known. In India, Mani 1989, Mani and Kurian 1953, Mani and Dubey 1972, Mani et al. 1973, and Mani et al. 1974 previously worked on this group. Other few notable contributions are by Joseph et al. 1973 (described 21 new species, 7 new sub species and made revisions of 43 known species of Oriental *Brachymeria*) and Narendran 1989 (described 4 new genera, 88 new species and made revisions of 37 known genera and 242 known species). Since then, work on Indian Chalcididae dwindled. In this paper, we document new records of Chalcididae from different states and union territories of India as outlined above.

## Materials and methods

Parasitoids were collected from Indian states Andhra Pradesh, Bihar, Karnataka, Himachal Pradesh, Manipur, Mizoram, Nagaland, and Tamil Nadu and union territories, Andaman & Nicobar Islands and Pudhucherry using yellow pan traps in different ecosystems. Specimens were dried using hexamethyldisilazane as described by Brown (1993) and card mounted using the standard procedure adopted by Noyes (1982). Specimens identified were deposited in the Parasitoid Taxonomy and Biocontrol laboratory, Department of Entomology, Annamalai University (EDAU).

## Taxon treatments

### Brachymeria albicrus

(Klug) 1834

#### Materials

- **Type status:** Other material. **Occurrence:** recordedBy: S. Manickavasagam; individualCount: 1; lifeStage: adult; **Location:** continent: Asia; country: India; countryCode: IND; stateProvince: Pudhucherry; **Identification:** identifiedBy: J. Gowri Prakash and S. Manickavasagam; **Event:** samplingProtocol: Yellow pan trap; eventDate: 03/13/2011; **Record Level:** institutionID: Department of Entomology, Annamalai University; institutionCode: EDAU

#### Distribution

This species is so far known from Odisha and Tamil Nadu (Noyes 2015) and is a new record for Pudhucherry (Fig. 1).

### Brachymeria alternipes

(Walker) 1871

#### Materials

- **Type status:** Other material. **Occurrence:** recordedBy: Not known; individualCount: 12; lifeStage: adult; **Location:** continent: Asia; country: India; countryCode: IND; stateProvince: Himachal Pradesh; **Identification:** identifiedBy: J. Gowri Prakash and S. Manickavasagam; **Event:** samplingProtocol: Yellow pan trap; eventDate: 09/23/1981; **Record Level:** institutionID: Department of Entomology, Annamalai University; institutionCode: EDAU

#### Distribution

*B. alternipes* is so far known from Arunachal Pradesh (Sheela et al. 2003), Tamil Nadu and Manipur (Narendran 1989) and is a new record for Himachal Pradesh (Fig. 2).

### Brachymeria apicicornis

(Cameron) 1911

#### Materials

- **Type status:** Other material. **Occurrence:** recordedBy: N. Gowthaman; individualCount: 13; lifeStage: adult; **Location:** continent: Asia; country: India; countryCode: IND; stateProvince: Tamil Nadu; **Identification:** identifiedBy: J. Gowri Prakash and S. Manickavasagam; **Event:** samplingProtocol: Yellow pan trap; eventDate: 08/12/2009; **Record Level:** institutionID: Department of Entomology, Annamalai University; institutionCode: EDAU
- **Type status:** Other material. **Occurrence:** recordedBy: S. Sowmiya; individualCount: 17; lifeStage: adult; **Location:** continent: Asia; country: India; countryCode: IND; stateProvince: Karnataka; **Identification:** identifiedBy: J. Gowri Prakash and S. Manickavasagam; **Event:** samplingProtocol: Yellow pan trap; eventDate: 09/24/2014; **Record Level:** institutionID: Department of Entomology, Annamalai University; institutionCode: EDAU
- **Type status:** Other material. **Occurrence:** recordedBy: S. Manickavasagam; individualCount: 1; lifeStage: adult; **Location:** continent: Asia; country: India; countryCode: IND; stateProvince: Pudhucherry; **Identification:** identifiedBy: J. Gowri Prakash and S. Manickavasagam; **Event:** samplingProtocol: Yellow pan trap; eventDate: 03/13/2011; **Record Level:** institutionID: Department of Entomology, Annamalai University; institutionCode: EDAU

#### Distribution

*B. apicicornis* is so far known from Kerala (Narendran 1989) and is a new record for Tamil Nadu and Karnataka (Fig. 3).

### Brachymeria euploeae

(Westwood) 1837

#### Materials

- **Type status:** Other material. **Occurrence:** recordedBy: Sophis Singh; individualCount: 20; lifeStage: adult; **Location:** continent: Asia; country: India; countryCode: IND; stateProvince: Manipur; **Identification:** identifiedBy: J. Gowri Prakash and S. Manickavasagam; **Event:** samplingProtocol: Yellow pan trap; eventDate: 02/18/2014; **Record Level:** institutionID: Department of Entomology, Annamalai University; institutionCode: EDAU
- **Type status:** Other material. **Occurrence:** recordedBy: Sophis Singh; individualCount: 5; lifeStage: adult; **Location:** continent: Asia; country: India; countryCode: IND; stateProvince: Mizoram; **Identification:** identifiedBy: J. Gowri Prakash and S. Manickavasagam; **Event:** samplingProtocol: Yellow pan trap; eventDate: 12/30/2014; **Record Level:** institutionID: Department of Entomology, Annamalai University; institutionCode: EDAU
- **Type status:** Other material. **Occurrence:** recordedBy: Sophis Singh; individualCount: 2; lifeStage: adult; **Location:** continent: Asia; country: India; countryCode: IND; stateProvince: Nagaland; **Identification:** identifiedBy: J. Gowri Prakash and S. Manickavasagam; **Event:** samplingProtocol: Yellow pan trap; eventDate: 12/20/2014; **Record Level:** institutionID: Department of Entomology, Annamalai University; institutionCode: EDAU

#### Distribution

**Figure 4.**
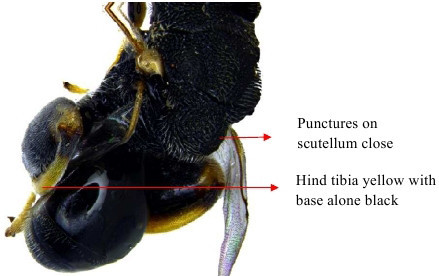
*Brachymeria euploeae* (Westwood)

This species so far known from Andaman and Nicobar island, Tripura, (Sheela et al. 2003) Arunachal Pradesh, Bihar, Haryana, Jharkhand, Karnataka, Madhya Pradesh, Manipur, Odisha, Punjab, Tamil Nadu, Uttar Pradesh, Uttarkhand and West Bengal (Narendran 1989) and is a new record for Manipur, Mizoram and Nagaland (Fig. 4).

### Brachymeria excarinata

Gahan 1925

#### Materials

- **Type status:** Other material. **Occurrence:** recordedBy: Abhinav Kumar; individualCount: 1; lifeStage: adult; **Location:** continent: Asia; country: India; countryCode: IND; stateProvince: Bihar; **Identification:** identifiedBy: J. Gowri Prakash and S. Manickavasagam; **Event:** samplingProtocol: Yellow pan trap; eventDate: 01/27/2015; **Record Level:** institutionID: Department of Entomology, Annamalai University; institutionCode: EDAU
- **Type status:** Other material. **Occurrence:** recordedBy: S. Manickavasagam and A. Ramesh Kumar; individualCount: 2; lifeStage: adult; **Location:** continent: Asia; country: India; countryCode: IND; stateProvince: Andaman and Nicobar islands; **Identification:** identifiedBy: J. Gowri Prakash and S. Manickavasagam; **Event:** samplingProtocol: Yellow pan trap; eventDate: 05/21/2012; **Record Level:** institutionID: Department of Entomology, Annamalai University; institutionCode: EDAU

#### Distribution

**Figure 5.**
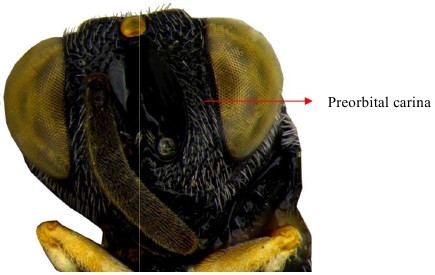
*Brachymeria excarinata* Gahan

*B. excarinata* is so far known from Gujarat, Karnataka, Kerala and Tamil Nadu (Joseph et al. 1973) and is a new record for Bihar and Andaman and Nicobar islands (Fig. 5).

### Brachymeria hearseyi

(Kirby) 1883

#### Materials

- **Type status:** Other material. **Occurrence:** recordedBy: Abhinav Kumar; individualCount: 1; lifeStage: adult; **Location:** continent: Asia; country: India; countryCode: IND; stateProvince: Bihar; **Identification:** identifiedBy: J. Gowri Prakash and S. Manickavasagam; **Event:** samplingProtocol: Yellow pan trap; eventDate: 07/08/2014; **Record Level:** institutionID: Department of Entomology, Annamalai University; institutionCode: EDAU
- **Type status:** Other material. **Occurrence:** recordedBy: Sophis Singh; individualCount: 20; lifeStage: adult; **Location:** continent: Asia; country: India; countryCode: IND; stateProvince: Manipur; **Identification:** identifiedBy: J. Gowri Prakash and S. Manickavasagam; **Event:** samplingProtocol: Yellow pan trap; eventDate: 02/18/2014; **Record Level:** institutionID: Department of Entomology, Annamalai University; institutionCode: EDAU
- **Type status:** Other material. **Occurrence:** recordedBy: Sophis Singh; individualCount: 5; lifeStage: adult; **Location:** continent: Asia; country: India; countryCode: IND; stateProvince: Mizoram; **Identification:** identifiedBy: J. Gowri Prakash and S. Manickavasagam; **Event:** samplingProtocol: Yellow pan trap; eventDate: 12/13/2014; **Record Level:** institutionID: Department of Entomology, Annamalai University; institutionCode: EDAU
- **Type status:** Other material. **Occurrence:** recordedBy: Sophis Singh; individualCount: 4; lifeStage: adult; **Location:** continent: Asia; country: India; countryCode: IND; stateProvince: Nagaland; **Identification:** identifiedBy: J. Gowri Prakash and S. Manickavasagam; **Event:** samplingProtocol: Yellow pan trap; eventDate: 12/22/2014; **Record Level:** institutionID: Department of Entomology, Annamalai University; institutionCode: EDAU

#### Distribution

*B. hearseyi* is so far known from Andaman and Nicobar Islands (Sheela et al. 2003) and West Bengal (Joseph et al. 1973) and is a new record for Bihar (Fig. 6).

### Brachymeria megaspila

(Cameron) 1907

#### Materials

- **Type status:** Other material. **Occurrence:** recordedBy: T. Krishna Chaitanya; individualCount: 1; lifeStage: adult; **Location:** continent: Asia; country: India; countryCode: IND; stateProvince: Andhra Pradesh; **Identification:** identifiedBy: J. Gowri Prakash and S. Manickavasagam; **Event:** samplingProtocol: Yellow pan trap; eventDate: 03/20/2010; **Record Level:** institutionID: Department of Entomology, Annamalai University; institutionCode: EDAU

#### Distribution

**Figure 7.**
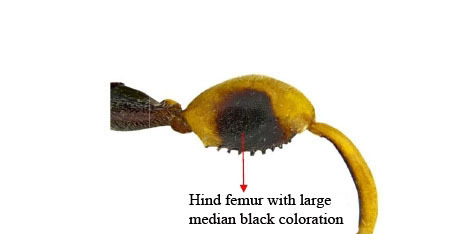
*Brachymeria megaspila* (Cameron)

*B. megaspila* is so far known from Assam, Kerala and Madhya Pradesh (Narendran 1989) and is a new record for Andhra Pradesh (Fig. 7).

### Brachymeria minuta

(Linnaeus) 1767

#### Materials

- **Type status:** Other material. **Occurrence:** recordedBy: T. Krishna Chaitanya; individualCount: 2; lifeStage: adult; **Location:** continent: Asia; country: India; countryCode: IND; stateProvince: Andhra Pradesh; **Identification:** identifiedBy: J. Gowri Prakash and S. Manickavasagam; **Event:** samplingProtocol: Yellow pan trap; eventDate: 03/20/2010; **Record Level:** institutionID: Department of Entomology, Annamalai University; institutionCode: EDAU

#### Distribution

**Figure 8.**
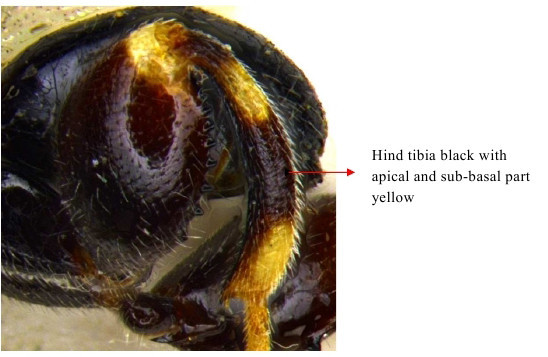
*Brachymeria minuta* (Linnaeus)

This species is so far known from Karnataka, Sikkim, Tamil Nadu, Uttar Pradesh and West Bengal (Narendran 1989) and is a new record for Andhra Pradesh (Fig. 8).

### Brachymeria podagrica

(Fabricius) 1787

#### Materials

- **Type status:** Other material. **Occurrence:** recordedBy: Abhinav Kumar; individualCount: 2; lifeStage: adult; **Location:** continent: Asia; country: India; countryCode: IND; stateProvince: Bihar; **Identification:** identifiedBy: J. Gowri Prakash, S. Manickavasagam and R.Kanagarajan; **Event:** samplingProtocol: Yellow pan trap; eventDate: 01/27/2015; **Record Level:** institutionID: Department of Entomology, Annamalai University; institutionCode: EDAU
- **Type status:** Other material. **Occurrence:** recordedBy: S. Manickavasagam and A. Ramesh kumar; individualCount: 3; lifeStage: adult; **Location:** continent: Asia; country: India; countryCode: IND; stateProvince: Andaman and Nicobar islands; **Identification:** identifiedBy: J. Gowri Prakash, S. Manickavasagam and R.Kanagarajan; **Event:** samplingProtocol: Yellow pan trap; eventDate: 05/21/2012; **Record Level:** institutionID: Department of Entomology, Annamalai University; institutionCode: EDAU

#### Distribution

**Figure 9.**
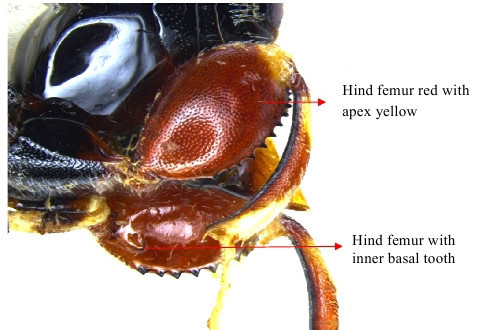
*Brachymeria podagrica* (Fabricius)

*B. podagrica* is so far known from Madhya Pradesh, Sikkim, Tamil Nadu and Tripura (Joseph et al. 1973) and is a new record for Bihar and Andaman and Nicobar islands (Fig. 9).

### Dirhinus anthracia

Walker 1846

#### Materials

- **Type status:** Other material. **Occurrence:** recordedBy: J. Gowri Prakash; individualCount: 40; lifeStage: adult; **Location:** continent: Asia; country: India; countryCode: IND; stateProvince: Tamil Nadu; **Identification:** identifiedBy: J. Gowri Prakash and S. Manickavasagam; **Event:** samplingProtocol: Yellow pan trap; eventDate: 2014-04-04 and 2014-07-29; **Record Level:** institutionID: Department of Entomology, Annamalai University; institutionCode: EDAU
- **Type status:** Other material. **Occurrence:** recordedBy: S. Manickavasagam; individualCount: 1; lifeStage: adult; **Location:** continent: Asia; country: India; countryCode: IND; stateProvince: Pudhucherry; **Identification:** identifiedBy: J. Gowri Prakash and S. Manickavasagam; **Event:** samplingProtocol: Yellow pan trap; eventDate: 03/13/2011; **Record Level:** institutionID: Department of Entomology, Annamalai University; institutionCode: EDAU
- **Type status:** Other material. **Occurrence:** recordedBy: T. Krishna Chaitanya; individualCount: 1; lifeStage: adult; **Location:** continent: Asia; country: India; countryCode: IND; stateProvince: Andhra Pradesh; **Identification:** identifiedBy: J. Gowri Prakash and S. Manickavasagam; **Event:** samplingProtocol: Yellow pan trap; eventDate: 01/06/2011; **Record Level:** institutionID: Department of Entomology, Annamalai University; institutionCode: EDAU
- **Type status:** Other material. **Occurrence:** recordedBy: Abhinav Kumar; individualCount: 6; lifeStage: adult; **Location:** continent: Asia; country: India; countryCode: IND; stateProvince: Bihar; **Identification:** identifiedBy: J. Gowri Prakash and S. Manickavasagam; **Event:** samplingProtocol: Yellow pan trap; eventDate: 02/22/2014; **Record Level:** institutionID: Department of Entomology, Annamalai University; institutionCode: EDAU
- **Type status:** Other material. **Occurrence:** recordedBy: Sophis singh; individualCount: 1; lifeStage: adult; **Location:** continent: Asia; country: India; countryCode: IND; stateProvince: Mizoram; **Identification:** identifiedBy: J. Gowri Prakash and S. Manickavasagam; **Event:** samplingProtocol: Yellow pan trap; eventDate: 12/30/2014; **Record Level:** institutionID: Department of Entomology, Annamalai University; institutionCode: EDAU

#### Distribution

*D. anthracia* is so far known from Madhya Pradesh, Manipur, Punjab, Tripura and Uttar Pradesh (Narendran 1989) and is a new record for Tamil Nadu, Pudhucherry, Andhra Pradesh, Bihar and Mizoram (Fig. 10).

### Dirhinus auratus

Ashmead 1905

#### Materials

- **Type status:** Other material. **Occurrence:** recordedBy: S. Manickavasagam; individualCount: 3; lifeStage: adult; **Location:** continent: Asia; country: India; countryCode: IND; stateProvince: Pudhucherry; **Identification:** identifiedBy: J. Gowri Prakash and S. Manickavasagam; **Event:** samplingProtocol: Yellow pan trap; eventDate: 03/13/2011; **Record Level:** institutionID: Department of Entomology, Annamalai University; institutionCode: EDAU
- **Type status:** Other material. **Occurrence:** recordedBy: T. Krishna Chaitanya; individualCount: 7; lifeStage: adult; **Location:** continent: Asia; country: India; countryCode: IND; stateProvince: Andhra Pradesh; **Identification:** identifiedBy: J. Gowri Prakash and S. Manickavasagam; **Event:** samplingProtocol: Yellow pan trap; eventDate: 10/03/2010; **Record Level:** institutionID: Department of Entomology, Annamalai University; institutionCode: EDAU
- **Type status:** Other material. **Occurrence:** recordedBy: Abhinav Kumar; individualCount: 2; lifeStage: adult; **Location:** continent: Asia; country: India; countryCode: IND; stateProvince: Bihar; **Identification:** identifiedBy: J. Gowri Prakash and S. Manickavasagam; **Event:** samplingProtocol: Yellow pan trap; eventDate: 04/22/2014; **Record Level:** institutionID: Department of Entomology, Annamalai University; institutionCode: EDAU
- **Type status:** Other material. **Occurrence:** recordedBy: Sophis Singh; individualCount: 4; lifeStage: adult; **Location:** continent: Asia; country: India; countryCode: IND; stateProvince: Manipur; **Identification:** identifiedBy: J. Gowri Prakash and S. Manickavasagam; **Event:** samplingProtocol: Yellow pan trap; eventDate: 11/29/2014; **Record Level:** institutionID: Department of Entomology, Annamalai University; institutionCode: EDAU
- **Type status:** Other material. **Occurrence:** recordedBy: N. Gowthaman and S. Palanivel; individualCount: 14; lifeStage: adult; **Location:** continent: Asia; country: India; countryCode: IND; stateProvince: Tamil Nadu; **Identification:** identifiedBy: J. Gowri Prakash and S. Manickavasagam; **Event:** samplingProtocol: Yellow pan trap; eventDate: 09/21/2014; **Record Level:** institutionID: Department of Entomology, Annamalai University; institutionCode: EDAU
- **Type status:** Other material. **Occurrence:** recordedBy: Abhinav Kumar; individualCount: 2; lifeStage: adult; **Location:** continent: Asia; country: India; countryCode: IND; stateProvince: Bihar; **Identification:** identifiedBy: J. Gowri Prakash and S. Manickavasagam; **Event:** samplingProtocol: Yellow pan trap; eventDate: 01/07/2015; **Record Level:** institutionID: Department of Entomology, Annamalai University; institutionCode: EDAU

#### Distribution

**Figure 11.**
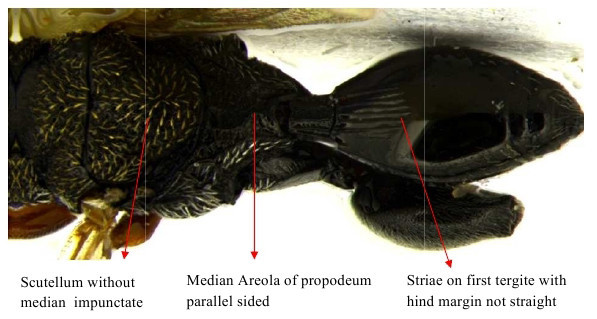
*Dirhinus auratus* Ashmead

This species is so far known from Delhi, Karnataka, Kerala, Madhya Pradesh, Punjab, Tamil Nadu, Uttar Pradesh and West Bengal (Noyes 2015) and is a new record for Pudhucherry, Andhra Pradesh, Bihar and Manipur (Fig. 11).

### Dirhinus bakeri

(Crawford) 1915

#### Materials

- **Type status:** Other material. **Occurrence:** recordedBy: S. Palanivel; individualCount: 3; lifeStage: adult; **Location:** continent: Asia; country: India; countryCode: IND; stateProvince: Tamil Nadu; **Identification:** identifiedBy: J. Gowri Prakash and S. Manickavasagam; **Event:** samplingProtocol: Yellow pan trap; eventDate: 03/02/2013; **Record Level:** institutionID: Department of Entomology, Annamalai University; institutionCode: EDAU

#### Distribution

*D. bakeri* is so far known from Delhi, Karnataka, Kerala, Madhya Pradesh, Odisha, Tripura, Uttar Pradesh and West Bengal (Noyes 2015) and is a new record for Tamil Nadu (Fig. 12).

### Dirhinus deplanatus

Boucek & Narendran 1981

#### Materials

- **Type status:** Other material. **Occurrence:** recordedBy: M. Ayyam Perumal, S. Palanivel and R.Kanagarajan; individualCount: 6; lifeStage: adult; **Location:** continent: Asia; country: India; countryCode: IND; stateProvince: Tamil Nadu; **Identification:** identifiedBy: J. Gowri Prakash and S. Manickavasagam; **Event:** samplingProtocol: Yellow pan trap; eventDate: 2014-02-22 and 2014-07-29; **Record Level:** institutionID: Department of Entomology, Annamalai University; institutionCode: EDAU

#### Distribution

**Figure 13.**
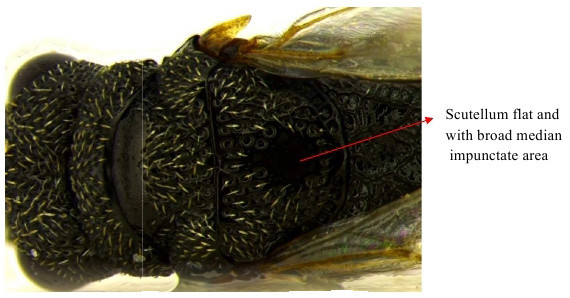
*Dirhinus deplanatus* Boucek & Narendran

*D. deplanatus* is so far known from Bihar, Delhi and Odisha (Narendran 1989) and is a new record for Tamil Nadu (Fig. 13).

### Dirhinus pilifer

Boucek & Narendran 1981

#### Materials

- **Type status:** Other material. **Occurrence:** recordedBy: N. Gowthaman and S. Palanivel; individualCount: 14; lifeStage: adult; **Location:** continent: Asia; country: India; countryCode: IND; stateProvince: Tamil Nadu; **Identification:** identifiedBy: J. Gowri Prakash and S. Manickavasagam; **Event:** samplingProtocol: Yellow pan trap; eventDate: 09/21/2014; **Record Level:** institutionID: Department of Entomology, Annamalai University; institutionCode: EDAU
- **Type status:** Other material. **Occurrence:** recordedBy: Abhinav Kumar; individualCount: 2; lifeStage: adult; **Location:** continent: Asia; country: India; countryCode: IND; stateProvince: Bihar; **Identification:** identifiedBy: J. Gowri Prakash and S. Manickavasagam; **Event:** samplingProtocol: Yellow pan trap; eventDate: 01/07/2015; **Record Level:** institutionID: Department of Entomology, Annamalai University; institutionCode: EDAU

#### Distribution

**Figure 14.**
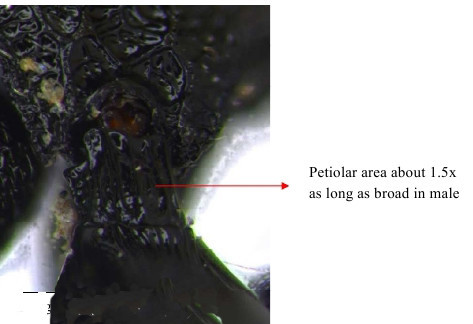
*Dirhinus pilifer* Boucek & Narendran

*D. pilifer* is so far known from Karnataka, Uttar Pradesh and West Bengal (Noyes 2015) and is a new record Tamil Nadu and Bihar (Fig. 14).

### Epitranus elongatulus

(Motschulsky) 1863

#### Materials

- **Type status:** Other material. **Occurrence:** recordedBy: N. Gowthaman and M. Ayyam Perumal; individualCount: 6; lifeStage: adult; **Location:** continent: Asia; country: India; countryCode: IND; stateProvince: Tamil Nadu; **Identification:** identifiedBy: J. Gowri Prakash and S. Manickavasagam; **Event:** samplingProtocol: Yellow pan trap; eventDate: 02/24/2014; **Record Level:** institutionID: Department of Entomology, Annamalai University; institutionCode: EDAU

#### Distribution

*E. elongatulus* is so far known from Delhi and Kerala (Narendran 1989) and is a new record for Tamil Nadu (Fig. 15).

### Epitranus erythrogaster

Cameron 1888

#### Materials

- **Type status:** Other material. **Occurrence:** recordedBy: S. Manickavasagam; individualCount: 11; lifeStage: adult; **Location:** continent: Asia; country: India; countryCode: IND; stateProvince: Pudhucherry; **Identification:** identifiedBy: J. Gowri Prakash and S. Manickavasagam; **Event:** samplingProtocol: Yellow pan trap; eventDate: 03/13/2011; **Record Level:** institutionID: Department of Entomology, Annamalai University; institutionCode: EDAU
- **Type status:** Other material. **Occurrence:** recordedBy: Abhinav Kumar; individualCount: 10; lifeStage: adult; **Location:** continent: Asia; country: India; countryCode: IND; stateProvince: Bihar; **Identification:** identifiedBy: J. Gowri Prakash and S. Manickavasagam; **Event:** samplingProtocol: Yellow pan trap; eventDate: 02/22/2014; **Record Level:** institutionID: Department of Entomology, Annamalai University; institutionCode: EDAU
- **Type status:** Other material. **Occurrence:** recordedBy: Sophis Singh; individualCount: 1; lifeStage: adult; **Location:** continent: Asia; country: India; countryCode: IND; stateProvince: Manipur; **Identification:** identifiedBy: J. Gowri Prakash and S. Manickavasagam; **Event:** samplingProtocol: Yellow pan trap; eventDate: 02/18/2014; **Record Level:** institutionID: Department of Entomology, Annamalai University; institutionCode: EDAU
- **Type status:** Other material. **Occurrence:** recordedBy: S. Manickavasagam and A. Rameshkumar; individualCount: 2; lifeStage: adult; **Location:** continent: Asia; country: India; countryCode: IND; stateProvince: Andaman and Nicobar islands; **Identification:** identifiedBy: J. Gowri Prakash and S. Manickavasagam; **Event:** samplingProtocol: Yellow pan trap; eventDate: 05/20/2012; **Record Level:** institutionID: Department of Entomology, Annamalai University; institutionCode: EDAU

#### Distribution

This species is so far known from Karnataka, Kerala, Maharashtra, Tamil Nadu, Uttar Pradesh and West Bengal (Boucek 1982) and is a new record for Pudhucherry, Bihar, Manipur and Andaman and Nicobar islands (Fig. 16).

### Epitranus observator

Walker 1862

#### Materials

- **Type status:** Other material. **Occurrence:** recordedBy: S. Palanivel and M. Ayyam Perumal; individualCount: 3; lifeStage: adult; **Location:** continent: Asia; country: India; countryCode: IND; stateProvince: Tamil Nadu; **Identification:** identifiedBy: J. Gowri Prakash and S. Manickavasagam; **Event:** samplingProtocol: Yellow pan trap; eventDate: 11/12/2014; **Record Level:** institutionID: Department of Entomology, Annamalai University; institutionCode: EDAU

#### Distribution

*E. observator* is so far known from Andaman and Nicobar island, Delhi, Gujarat, Karnataka, Odisha, Rajastan, Sikkim, Tripura, Uttar Pradesh and West Bengal (Boucek 1982) and is a new record for Tamil Nadu (Fig. 17).

### Psilochalcis carinigena

(Cameron) 1907

#### Materials

- **Type status:** Other material. **Occurrence:** recordedBy: Abhinav Kumar; individualCount: 1; lifeStage: adult; **Location:** continent: Asia; country: India; countryCode: IND; stateProvince: Bihar; **Identification:** identifiedBy: J. Gowri Prakash, S. Manickavasagam and R.Kanagarajan; **Event:** samplingProtocol: Yellow pan trap; eventDate: 01/22/2015; **Record Level:** institutionID: Department of Entomology, Annamalai University; institutionCode: EDAU
- **Type status:** Other material. **Occurrence:** recordedBy: S. Manickavasagam and A. Rameshkumar; individualCount: 4; lifeStage: adult; **Location:** continent: Asia; country: India; countryCode: IND; stateProvince: Andaman and Nicobar island; **Identification:** identifiedBy: J. Gowri Prakash, S. Manickavasagam and R.Kanagarajan; **Event:** samplingProtocol: Yellow pan trap; eventDate: 05/20/2012; **Record Level:** institutionID: Department of Entomology, Annamalai University; institutionCode: EDAU

#### Distribution

*P. carinigena* is so far known from Gujarat, Karnataka, Kerala, Madhya Pradesh, Tripura and West Bengal (Narendran 1989) and is a new record for Bihar and Andaman and Nicobar island (Fig. 18).

### Psilochalcis keralensis

Narendran 1989

#### Materials

- **Type status:** Other material. **Occurrence:** recordedBy: S. Manickavasagam; individualCount: 8; lifeStage: adult; **Location:** continent: Asia; country: India; countryCode: IND; stateProvince: Pudhucherry; **Identification:** identifiedBy: J. Gowri Prakash and S. Manickavasagam; **Event:** samplingProtocol: Yellow pan trap; eventDate: 03/13/2011; **Record Level:** institutionID: Department of Entomology, Annamalai University; institutionCode: EDAU
- **Type status:** Other material. **Occurrence:** recordedBy: S. Palanivel and N. Gowthaman; individualCount: 2; lifeStage: adult; **Location:** continent: Asia; country: India; countryCode: IND; stateProvince: Tamil Nadu; **Identification:** identifiedBy: J. Gowri Prakash and S. Manickavasagam; **Event:** samplingProtocol: Yellow pan trap; eventDate: 07/29/2014; **Record Level:** institutionID: Department of Entomology, Annamalai University; institutionCode: EDAU

#### Distribution

**Figure 19.**
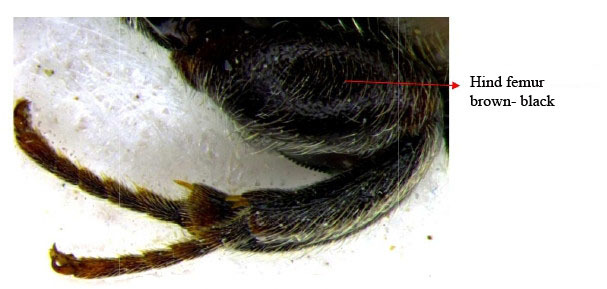
*Psilochalcis keralensis* Narendran

This species is so far known only from Kerala and Tripura (Narendran 1989) and is a new record for Tamil Nadu and Pudhucherry (Fig. 19).

### Psilochalcis soudanensis

(Steffan) 1951

#### Materials

- **Type status:** Other material. **Occurrence:** recordedBy: S. Palanivel and M. Ayyam Perumal; individualCount: 2; lifeStage: adult; **Location:** continent: Asia; country: India; countryCode: IND; stateProvince: Tamil Nadu; **Identification:** identifiedBy: J. Gowri Prakash and S. Manickavasagam; **Event:** samplingProtocol: Yellow pan trap; eventDate: 05/02/2014; **Record Level:** institutionID: Department of Entomology, Annamalai University; institutionCode: EDAU

#### Distribution

**Figure 20.**
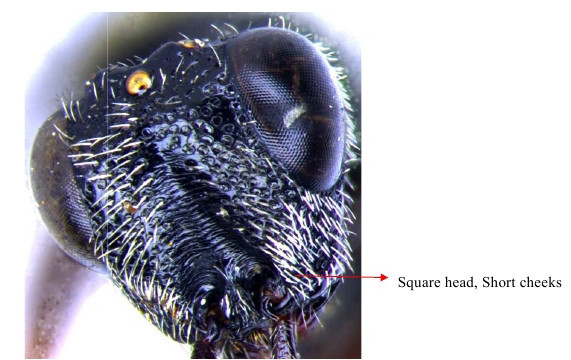
*Psilochalcis soudanensis* (Steffan)

This species is so far known from Andhra Pradesh, Delhi and Odisha (Narendran 1989) and is a new record for Tamil Nadu (Fig. 20).

## Supplementary Material

XML Treatment for Brachymeria albicrus

XML Treatment for Brachymeria alternipes

XML Treatment for Brachymeria apicicornis

XML Treatment for Brachymeria euploeae

XML Treatment for Brachymeria excarinata

XML Treatment for Brachymeria hearseyi

XML Treatment for Brachymeria megaspila

XML Treatment for Brachymeria minuta

XML Treatment for Brachymeria podagrica

XML Treatment for Dirhinus anthracia

XML Treatment for Dirhinus auratus

XML Treatment for Dirhinus bakeri

XML Treatment for Dirhinus deplanatus

XML Treatment for Dirhinus pilifer

XML Treatment for Epitranus elongatulus

XML Treatment for Epitranus erythrogaster

XML Treatment for Epitranus observator

XML Treatment for Psilochalcis carinigena

XML Treatment for Psilochalcis keralensis

XML Treatment for Psilochalcis soudanensis

## Figures and Tables

**Figure 1a.**
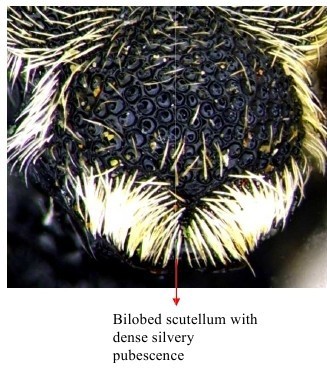
Scutellum dorsal view

**Figure 1b.**
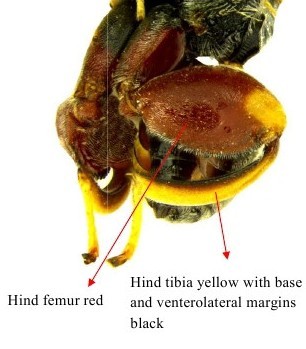
Hind leg

**Figure 2a.**
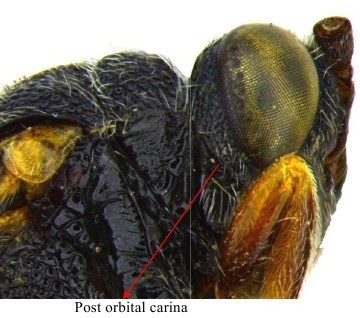
Head & part of mesosoma lateral view

**Figure 2b.**
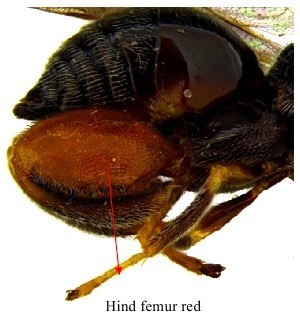
Gaster & hind leg

**Figure 3a.**
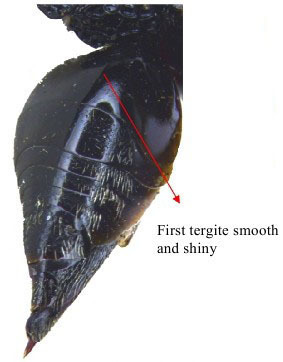
Gaster dorso lateral

**Figure 3b.**
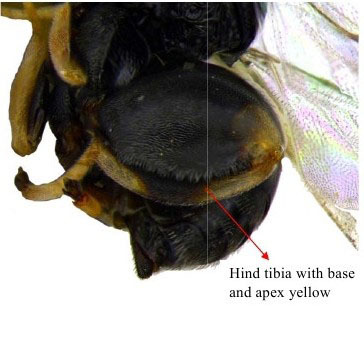
Hind leg

**Figure 6a.**
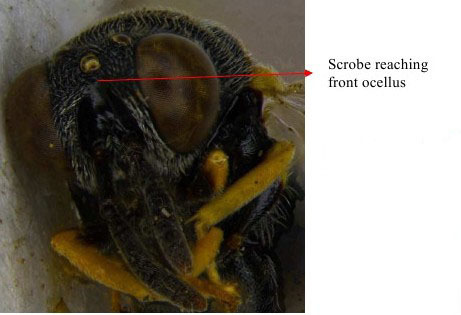
Head frontal showing scrobe

**Figure 6b.**
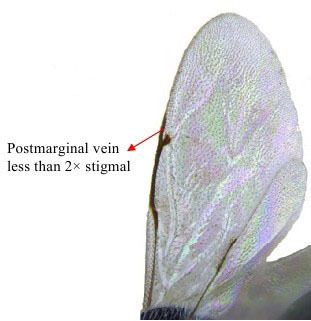
Fore wing

**Figure 10a.**
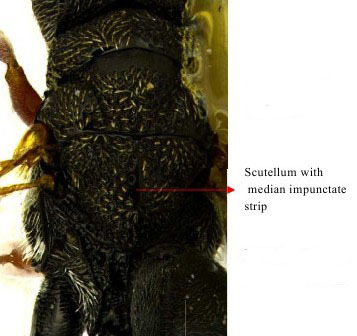
Part of mesosoma dorsal

**Figure 10b.**
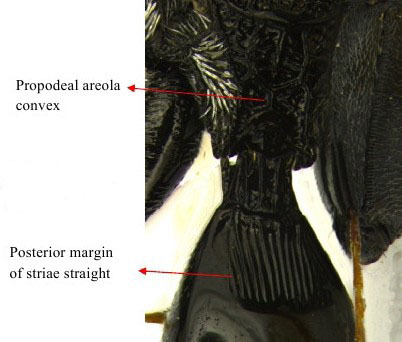
Propodeum, petiole and gastral segment 1

**Figure 12a.**
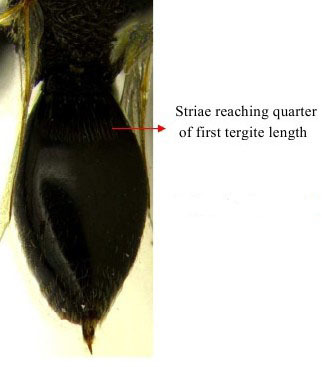
Mid part of soma

**Figure 12b.**
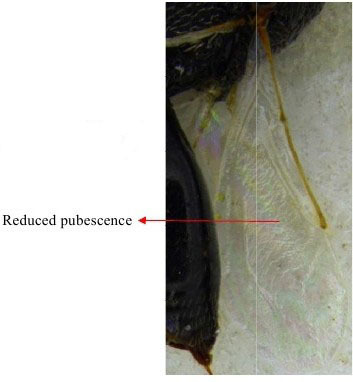
Fore wing

**Figure 15a.**
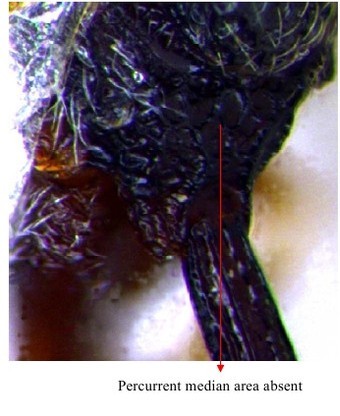
Propodeum & petiole

**Figure 15b.**
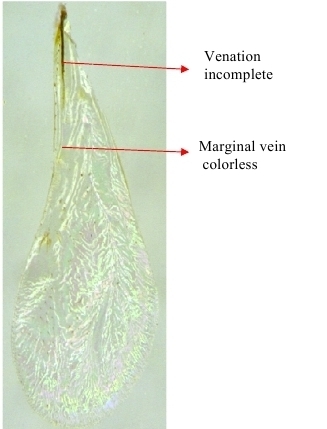
Fore wing

**Figure 16a.**
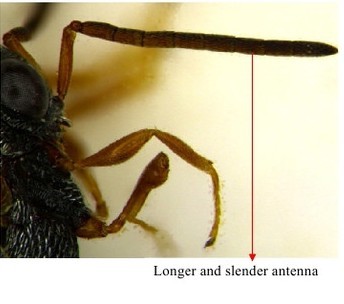
Antenna

**Figure 16b.**
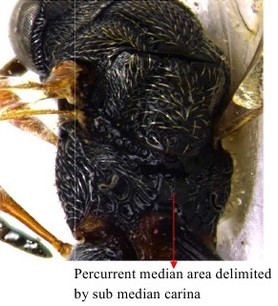
Mesosoma

**Figure 17a.**
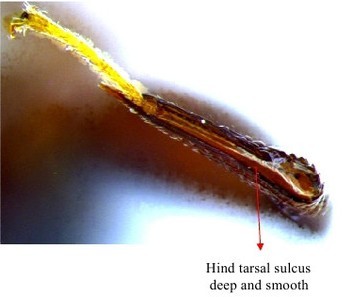
Hind tibia

**Figure 17b.**
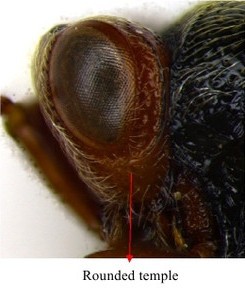
Head lateral

**Figure 18a.**
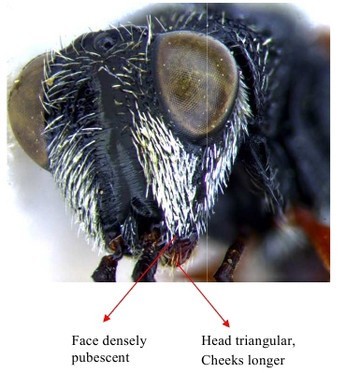
Head frontal

**Figure 18b.**
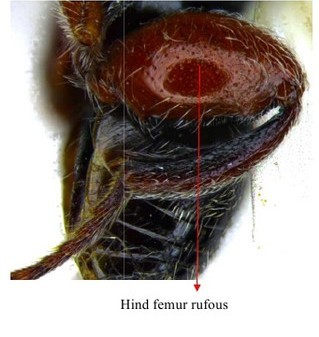
Hind leg
